# Two-dimensional assessments of civility and incivility at work

**DOI:** 10.1186/s40359-026-04927-2

**Published:** 2026-06-09

**Authors:** Dan Hasson, Cecilia Berlin, Karin Villaume

**Affiliations:** 1 Department of Global Public Health, Karolinska Institutet, PROCOME, Stockholm, 171 77 Sweden; 2 Mayo Clinic, Scottsdale, USA; 3 Department of Mechanical Engineering, Division of Design & Human Factors, Chalmers University of Technology, Gothenburg, Sweden

**Keywords:** Incivility, Civility, Assessment, Questionnaire, Two-dimensional, Coping, Psychosocial work environment

## Abstract

**Background:**

Civility and incivility are two multifaceted constructs that can be difficult to assess in a nuanced way. Previous studies have only utilized unidimensional assessments, which may have yielded partially misleading results and conclusions.

**Methods:**

The aims of the current mixed methods study were to investigate the 4-week prevalence of civility and incivility in a sample in the Swedish retail sector, and to assess if two-dimensional assessments can yield more nuanced interpretations of the civility and incivility constructs. The cross-sectional study included 1,014 employees out of which about 41% responded to the whole questionnaire and 59% answered it almost entirely or partially; approximately 50% responded to the civility and incivility questions presented in the current study.

**Results:**

The 4-week prevalence of incivility was approximately 62%, which is lower than in previous studies. Two-dimensional civility and incivility assessments and open-ended questionnaire responses demonstrate that these constructs can be interpreted in a more nuanced way. The notion that civility or incivility, mostly, or on average, is associated with positive or negative experiences does not necessarily mean that it applies for everyone.

**Conclusion:**

This insight becomes particularly important when assessments are used as a basis for efforts to systematically ensure good and healthy work environments. Scientifically, the results implicate the importance of using two-dimensional assessments for more accurate and nuanced conclusions. In occupational settings, dialogues need to be conducted to understand the true meaning of assessments to enable adequate actions based on accurate conclusions, rather than preconceived notions.

**Supplementary Information:**

The online version contains supplementary material available at 10.1186/s40359-026-04927-2.

## Background

Civility and incivility are two constructs that can have various meanings and can therefore be difficult to define and assess. According to the Merriam Webster dictionary, “incivility” is defined as “*the quality or state of being uncivil*” or “*a rude or discourteous act*”. Civility is described as “*civilized conduct*” or “*a polite expression*”. However, something that is perceived as civil or uncivil by one person is not necessarily experienced in the same way, or as equally problematic, by someone else. Rather, incivility seems to move along a continuum with behaviors ranging from unintentional (e.g., negative, clumsy and insensitive behaviors and gestures), to intentional (e.g., verbal or physical aggression, harassment, bullying, etcetera) [1, 2]. Thus, incivility is not necessarily intentional, but it often is when it comes to verbal or physical aggression that has higher intensity. Ultimately, it is the *experience* of a behavior that determines if it in fact was uncivil or not [3]. Whereas incivility is often described in terms of “disrespectful”, “rude”, “dysfunctional” or “unprofessional” behaviors, civility is instead more about empathetic, caring, friendly and polite behaviors. Descriptions of civility are reminiscent of what can be described as “common sense” and “etiquette” [4, 5]. According to Klein and Forni [4], civility is about showing that you care about the feelings and needs of others. Incivility could in other words be described as the opposite, i.e., rude or discourteous behaviors with disregard to others, irrespective of whether the act is intentional or unintentional [5]. Hence, according to Andersson and Pearson’s definition, “*Workplace incivility is low-intensity deviant behavior with ambiguous intent to harm the target*,* in violation of workplace norms for mutual respect. Uncivil behaviors are characteristically rude and discourteous*,* displaying lack of regard to others.*” [5].

There are apparent difficulties in conceptualizing workplace civility and incivility, and distinguish these concepts from other related constructs, such as counterproductive work behaviors [6], organizational citizenship behaviors [7], and related domains (e.g., aggression, bullying, harassment) [8]. For instance, Hershcovis [9] demonstrated that incivility, abusive supervision, bullying, social undermining, and interpersonal conflict exhibit similar correlations with outcomes. On the other hand, a meta-analytical review of 105 independent samples demonstrated that workplace incivility constitutes a valid and reliable construct [10]. They emanated from the original conceptualization of incivility entailing low-intensity and non-intrusive forms of mistreatment [5, 11]. So, despite that civility and incivility constructs are conceptualized differently in various studies, at least measures of incivility seem to exhibit sufficient levels of various forms of validity. Consequently, other related conceptualizations, such as counterproductive work behaviors [6], organizational citizenship behaviors [7], and other forms of workplace mistreatment (e.g., aggression, bullying, harassment, ostracism, abusive supervision, etc.), may well be related to or included in some civility and incivility definitions, conceptualizations and perceptions [8, 9].

So, the concepts of civility and incivility seem to entail or be strongly associated with several other related constructs [8–10]. Thus, in a broader sense, all forms of workplace mistreatment can be considered as uncivil, although various behaviors can be operationalized differently depending on the focus and specific definitions of different studies. As a result, the perceptions or consequences ultimately determine if behaviors are experienced or rated as civil or uncivil [12]. For instance, bringing cookies to your colleagues every now and then can be perceived as something pleasant, but doing so every day can instead be perceived as intrusive.

Incivility and disrespectful behaviors are common, antisocial behaviors at work [11, 13–15]. In several studies, Christine Porath and colleagues have reported about the overwhelming prevalence of workplace incivility and documented several negative consequences [16–20]. For instance, Porath and Pearson [20] report that 99% have witnessed incivility at work and 96% have been exposed to it themselves. Others report incivility incidence figures of 71% [11] and recent meta-analytic reviews have established prevalence rates of 75% [8, 12], which is about twice or more as much of the prevalence of other forms of workplace mistreatment [8]. Accordingly, most, if not all employees have apparently been subjected to or witnessed incivility at work. However, the prevalence figures of many studies may be over-estimated given that the retrospective ratings were made for the past year or five years. A more recent publication from the current sample demonstrated incivility prevalence of 62%, but the single question was posed in present tense and may thus represent an under-estimation [21]. Nevertheless, a multitude of health-, stress- and work-related associations were found among those reporting prevalence of incivility at work. Whereas incivility is systematically related to a wide array of negative health and work environment related outcomes, interventions to increase civility seem to yield profound positive effects on stress-health-, relationship- and work-related outcomes [22].

### Two-dimensional assessments

Despite civility and incivility seemingly being multifaceted constructs, previous studies have utilized unidimensional response formats. For instance, questions or statements are typically answered with response alternatives such as “Strongly Disagree” to “Strongly Agree” [23], or “Never” to “Daily” [24]. However, unidimensional response formats can be misleading when assessing multifaceted constructs [25]. Accordingly, even if frequent prevalence of civility is experienced as something positive by most, it may not be so for all. Some persons may rather be bothered by civility, feeling that it creates tense and superficial relationships, or that they have to behave in ways that are not perceived as genuine or authentic for them.

In such cases, a two-dimensional assessment approach can be more appropriate and may perhaps capture a more nuanced representation of the constructs [25]. Briefly, this approach utilizes completing questions with distinct response formats that indicate different aspects (here referred to as “dimensions”) of the same construct. Consequently, one can examine *both* how often something occurs and how satisfied or dissatisfied the respondent is with the outcome. As a result, the respondent provides both the frequency dimension of civil and uncivil behaviors, and their appraisal regarding if the selected frequency is perceived as a problem or not. Adding the appraisal dimension may be relevant theoretically since it may nuance the interpretation of civility and incivility ratings. This may in turn provide a better understanding of possible subgroups of individuals that, for various reasons, may not perceive these constructs in the same way that the majority does. In organizational practice such nuance is important to establish if interventions are needed or not and for whom.

To our knowledge, no previous studies have assessed civility and incivility in this way and only few previous studies have investigated two-dimensional assessments in work environment-related areas [25–27]. Wännström et al. (2008) [27] reported that the satisfaction dimension better predicts outcomes like work demands, but not leadership. Hasson et al. (2023) [25] also found that the satisfaction dimension of workload was a consistently superior predictor of health, wellbeing, recovery, stress and psychosocial work environment-related outcomes in several repeated assessments over two years. Only measuring satisfaction cannot however provide sufficient information about the nature of the construct itself. Given that self-reports, as well as satisfaction or dissatisfaction, to some extent are related to attitudes, personality or mood dispositions [28–32], and may not merely be an outcome, it can be difficult to know how to intervene without additional information. For instance, dissatisfaction with frequent incivility exposure would likely require other interventions than dissatisfaction with rare exposure.

It should be mentioned that satisfaction or dissatisfaction with ratings of a construct may not strictly represent a dimension of it. Appraisal may be influenced by several related or unrelated factors, such as personality, mood dispositions, etcetera, but also by organizational or situational circumstances. Satisfaction and dissatisfaction can also partly, fully, or not at all be an outcome of civility or incivility themselves. It may therefore be argued that the appraisal of civility or incivility do not constitute dimensions of them. However, the appraisal of a construct is likely also an inert dimension of it. The Sense of Humor Questionnaire [33, 34], for example, includes two dimensions, that is, the cognitive aspect of understanding humor, and the social dimension of liking it. The combination of these two dimensions constitutes a valid measure of the sense of humor construct. Satisfaction has previously not been suggested as a dimension of civility or incivility. However, it is certainly a related aspect that may be needed for accurate and proper assessment, as well as nuanced interpretation and understanding of results from scientific studies and organizational surveys.

Questions with two-dimensional response formats increase the precision and accuracy of the responses, in the sense that it nuances them significantly [25]. For example, it may turn out that some perceive themselves to often behave in a civil manner and are dissatisfied with it. A possible explanation for this could be that they for example would like to be able to speak their mind more often. The two-dimensional assessments have never been used to assess civility and incivility before. The results of this study will therefore enrich the research area both methodologically and possibly also in terms of more nuanced conclusions.

### Open-ended questions

While two-dimensional questions may account for two related perspectives of the same construct, open-ended questions can be used to seek an even more detailed and profound understanding of the results [35–37]. Using both two-dimensional and open-ended questions simultaneously enable a deeper and an enriched understanding of the results. This entails a mixed methods design with a Qual+QUAN structure, i.e., the qualitative component is supportive to the quantitative “main” data geared at complementarity [38]. Whereas the two-dimensional questions are analyzed quantitatively, the open-ended questions are analyzed qualitatively. This mixed methods approach may be useful to obtain broader and better understanding of the civility and incivility constructs.

In summary, incivility involves common, antisocial and intrusive behaviors at work, and the constructs of civility and incivility seem to be conceptualized in various ways [8–10, 12, 15]. However, to our knowledge, no previous study has investigated neither civility nor incivility using a two-dimensional approach that accounts for the inherent complexity of these constructs. To properly assess the feasibility of such a novel approach there is a need to investigate three specific aspects, i.e., prevalence, pedagogy and interpretability. For face validity, such measure should emanate in similar prevalence figures as previous studies. Visualizations of the two-dimensional outcomes need to be produced to assess the pedagogic usability of the presentation and its possible capacity to present more nuanced interpretations of the results. To obtain a deeper understanding of such interpretations, open-ended questions could be used to corroborate or explain various outcomes of the two-dimensional assessments. Such studies could provide a more profound and nuanced understanding of civility and incivility, as well as improve the validity of assessments. Consequently, this would facilitate for both scientists and practitioners to emanate from accurate conclusions and not preconceived notions in assessments and interventions.

### Aims

The aims of the current study are to:

- Assess the past 4-week prevalence and frequency of civility and incivility in a sample from the Swedish retail sector.
- Use a novel, two-dimensional assessment approach to investigate possibly more nuanced interpretations of the constructs.
- Investigate if responses to open-ended questions can provide explanations for the possible unexpected two-dimensional combinations of the civility and incivility constructs.

## Methods

The current, cross-sectional study was conducted between 2019 and 2021. A mixed method, single sample design with more weight on quantitative than on qualitative data was used. This design was selected since both the quantitative and the qualitative data complement each other [39] to increase understanding.

### Participants and procedures

**Fig. 1:**
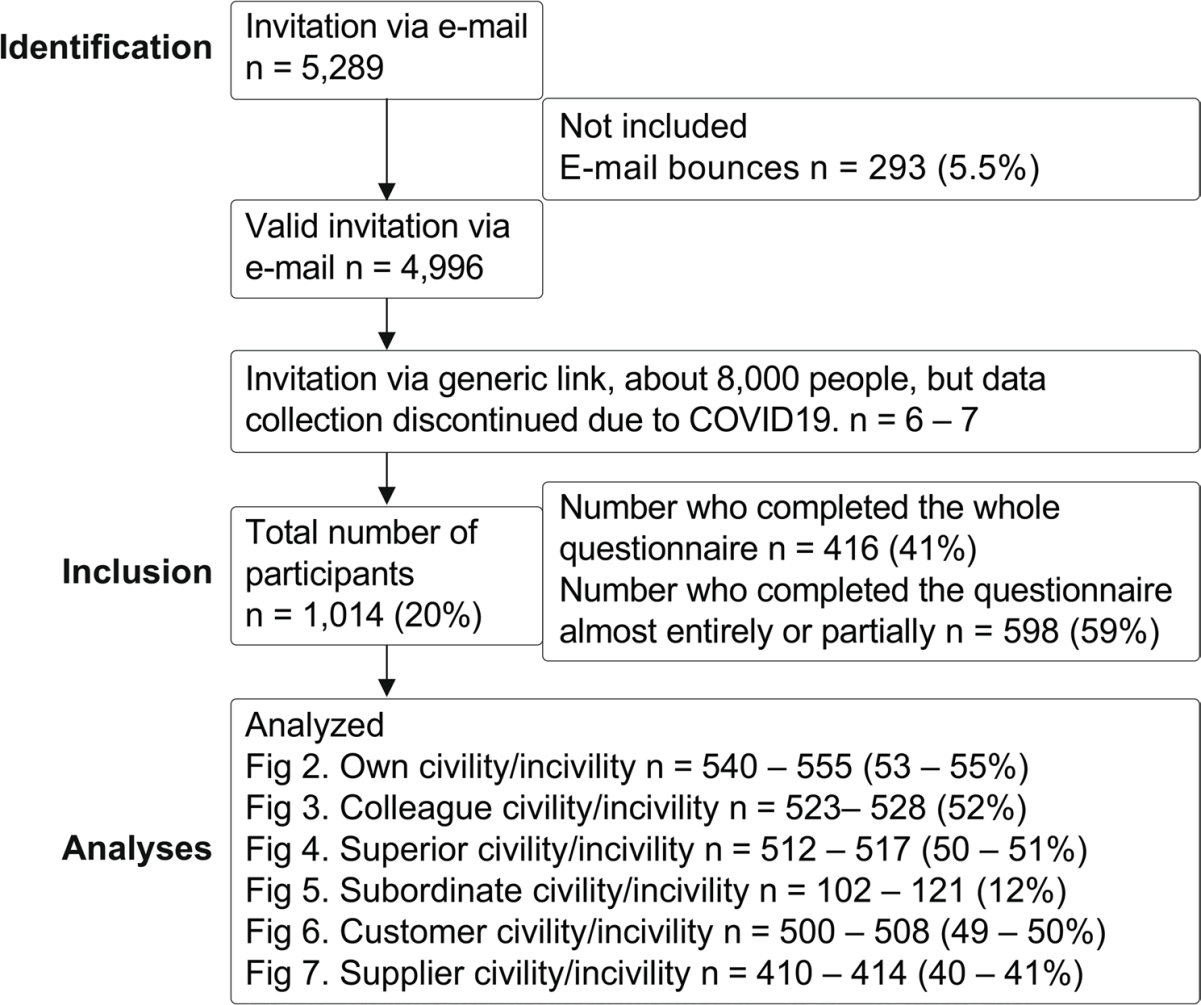
Flow of the participants

The participants and procedures have been described in more detail elsewhere [21]. Briefly, 5,289 employees in three retail companies were invited to participate (see Fig. 1 describing the flow of the participants). Employees at two of the companies were invited by email, whereas employees at the third company were invited to participate via a generic link that was posted in the lunchroom. A total of 1,014 (20%) individuals enrolled in the study, out of which 416 participants fully completed the questionnaire, and 598 completed it almost entirely or partially. 22% of those who started to respond or completed the survey were managers. One deviation relevant to the present study was that one person responded to over half the questionnaire but was removed from the dataset since it was obvious that all answers had been deliberately distorted and submitted with malicious intent.

### Possible explanations for the low response rates

The study generated far fewer participants than expected and there are several reasons for this, some of which are known to the authors, while others are not. For instance, it was not possible for the study participants to respond to the questionnaire during working hours. Thus, they primarily responded in their spare time. The web-based questionnaire was extensive and took between approximately 25 and 45 min to complete. Perhaps a shorter questionnaire would have yielded increased participation and completion, a notion that was indicated by some open-ended responses at the end of the questionnaire. At the same time, 1,014 individuals did in fact participate, indicating commitment and interest among the enrolled study participants. This notion is supported by some participants stressing the importance of the topic in their comments. Lastly, the study was conducted within retail, a sector that was significantly impacted by the COVID-19 pandemic in the spring of 2020. This may certainly have affected the response rates since stores limited opening hours and staff were laid off. To increase response rates, 7–8 reminders were distributed, and all reminders yielded additional responses.

### Questionnaire

The web-based questionnaire included approximately 300 items covering a multitude of aspects, such as civility, incivility, health and wellbeing, and symptoms of long-term stress. Other relevant aspects, such as personality traits, coping strategies, and several indicators of the psychosocial work environment (e.g., demand, control, support), etcetera, were also assessed. The present study uses selected closed (quantitative), and open (qualitative) items described below.

#### Two-dimensional assessment of civility and incivility

Initially, the authors compiled the following straightforward item about incivility:

*“Incivility is defined here as disrespectful behavior such as being impolite, ruthless or rude to someone else. Is there incivility at your workplace?”*

The response alternatives were: *Yes/No/Don’t know*, and the results from analyzing this single item in relation to other outcomes has been reported elsewhere and will therefore not be further addressed here [21]. It is still relevant to mention this questions, since those who responded “Yes”, received open-ended follow-up questions (see Appendix A) that have not been presented before and are analyzed here. Firstly, the four separate items: “*How do you / your immediate colleagues / immediate superior / organization deal with incivility when it occurs?*”. Thereafter, the following open-ended questions were posed: *“Does the organization’s dealing with incivility lead to any consequences*,* and if so which ones?”*,* “Can you give any examples of how you/colleagues/superior/organization have dealt with incivility in a good way?”*,* “How would you like incivility to be dealt with by you/colleagues/superior/organization at your workplace?”*. The last closed item/s were: *“Are there guidelines in the organization about incivility?”*, *(Yes/No/Don’t know)* and, if Yes: *“To what extent are they abided?”*,* (Not at all/To some extent/Quite a lot/Completely)*.

**Fig. 2:**
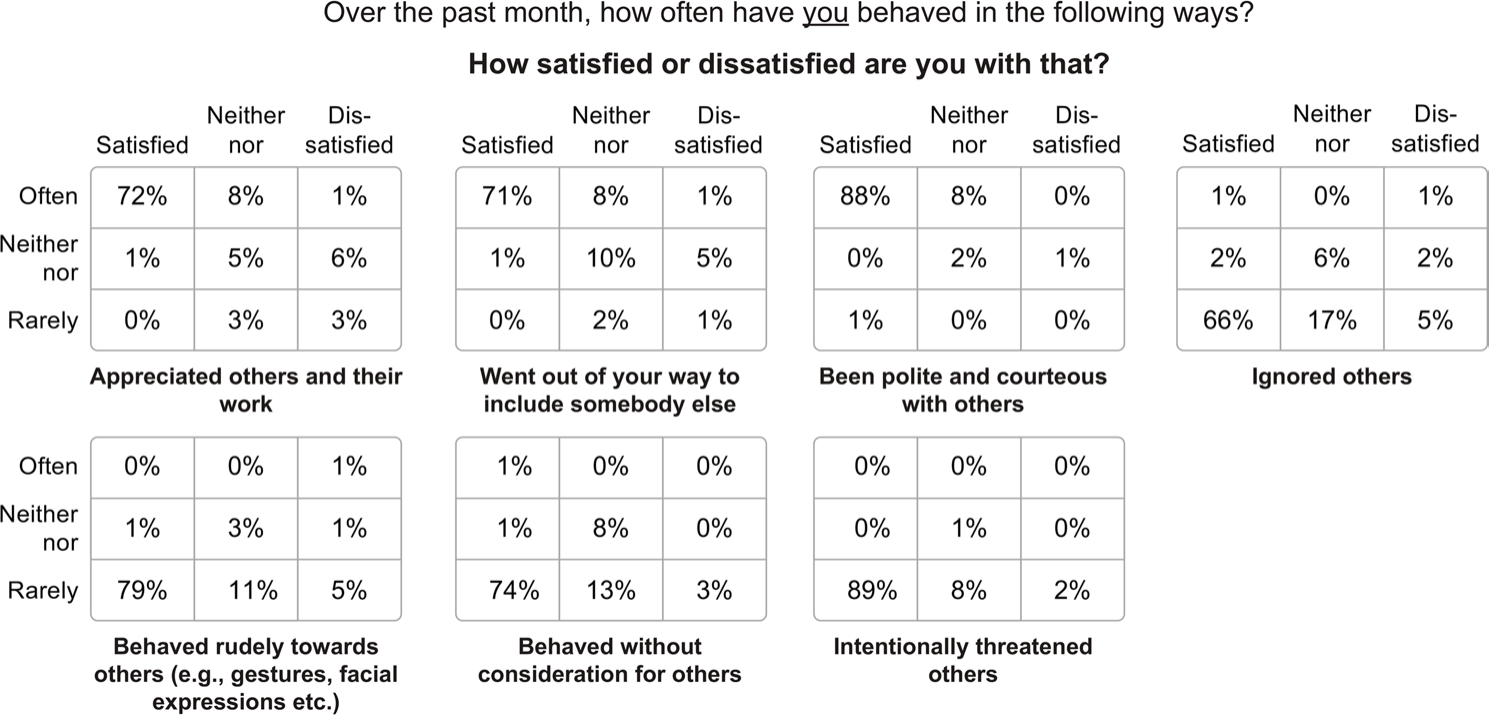
Two-dimensional assessments (crosstabs) of own civil and uncivil behaviors towards others, as well as satisfaction and dissatisfaction with the frequency of behaviors (*n* = 540–555)

**Fig. 3:**
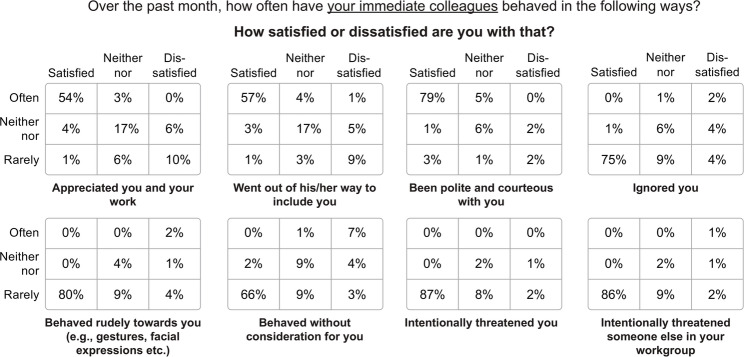
Two-dimensional assessments (crosstabs) of immediate colleagues’ civil and uncivil behaviors towards others, as well as satisfaction and dissatisfaction with the frequency of behaviors (*n* = 523 – 528)

**Fig. 4:**
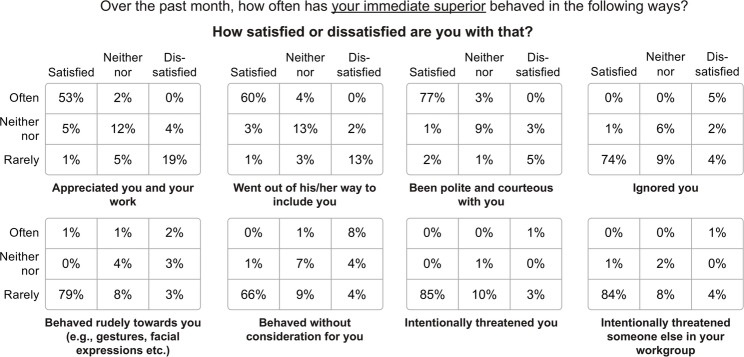
Two-dimensional assessments (crosstabs) of immediate superior’s civil and uncivil behaviors towards others, as well as satisfaction and dissatisfaction with the frequency of behaviors (*n* = 512 – 517)

**Fig. 5:**
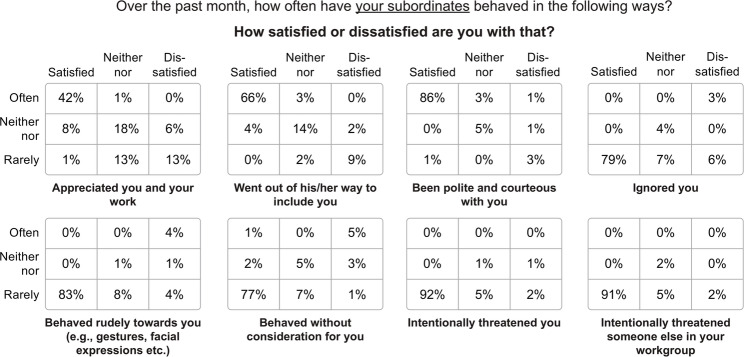
Two-dimensional assessments (crosstabs) of subordinates’ civil and uncivil behaviors towards others, as well as satisfaction and dissatisfaction with the frequency of behaviors (*n* = 120 – 121)

**Fig. 6:**
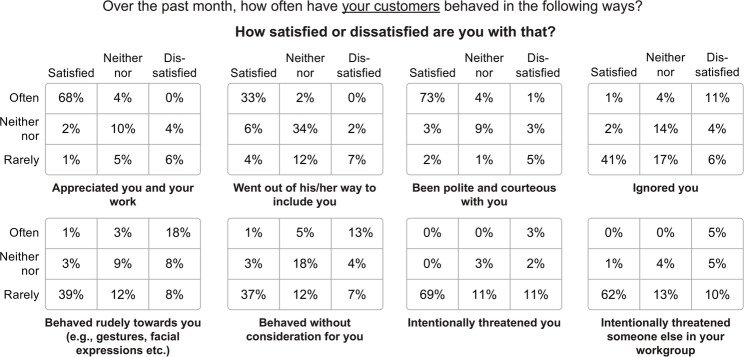
Two-dimensional assessments (crosstabs) of customers’ civil and uncivil behaviors towards others, as well as satisfaction and dissatisfaction with the frequency of behaviors (*n* = 500 – 508)

**Fig. 7:**
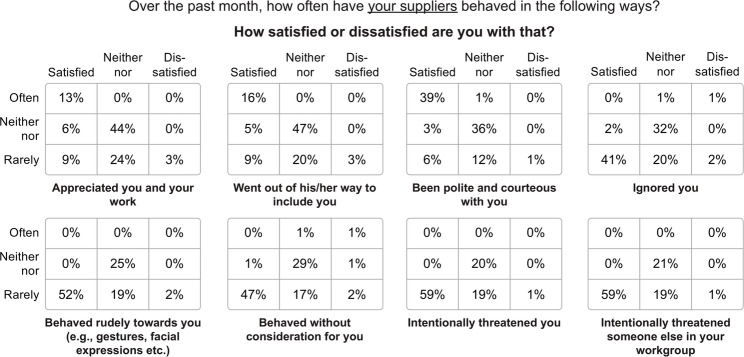
Two-dimensional assessments (crosstabs) of suppliers’ civil and uncivil behaviors towards others, as well as satisfaction and dissatisfaction with the frequency of behaviors (*n* = 410 – 414)

To assess civility and incivility in a novel, more nuanced way, two-dimensional questions were utilized to examine both how often certain behaviors occurred, and how satisfied or dissatisfied they were with it (see Figs. 2, 3, 4, 5, 6 and 7). The questionnaire investigated various civil and uncivil behaviors, and the participants were asked about own behaviors as well as behaviors of colleagues and immediate superior. For those who stated that they had interactions with customers, suppliers and/or subordinate staff, corresponding two-dimensional items were added to assess these perspectives as well.

Participants were asked to consider the *past four weeks* when responding to the questions and indicate how frequent different behaviors occurred. They were also asked how satisfied or dissatisfied they were with the frequency of each behavior. The original questions were derived from the Social Encounters Questionnaire [24] and the Straight Forward Incivility Scale [40, 41]. With permission from the aforementioned authors, the questions and response formats were slightly modified, i.e., revised to have five response alternatives instead of seven (see below), and completed with the second dimension about satisfaction and dissatisfaction. The questions were also translated into Swedish by the authors and proofread by other colleagues for face validity. Thus, most civility and incivility items were derived from the Social Encounters Questionnaire, and then further developed. The questions, here exemplified with own behaviors, were formulated as follows: *“Over the past 4 weeks*,* how often have you behaved in the following ways? Appreciated others and their work; Went out of your way to include somebody else; Been polite and courteous with others; Ignored others; Behaved rudely towards others (e.g.*,* gestures*,* facial expressions etc.); Behaved without consideration for others; Intentionally threatened others”*. Response alternatives ranged from “Very Seldom/Never” to “Very often/Always”. The second dimension of these questions asked: *“How satisfied or dissatisfied are you with that?”*, with responses ranging from “Very dissatisfied” to “Very satisfied”.

### Data-analytic approach

Percentages for all variables were plotted in crosstabulations (hereafter referred to as crosstabs or two-dimensional response combinations), with one dimension depicting frequency and the other satisfaction with selected frequency. The two highest and two lowest response alternatives were merged and renamed in order to create pedagogic crosstabs (3 × 3 graphs). For example, the response alternatives “Very Seldom and Never” were merged and named “Rarely” in the crosstabs (see Figs. 2, 3, 4, 5, 6 and 7).

Since qualitative data were collected using open-ended free-text responses, there was an opportunity to gain more detailed insight into the quantitative results, since qualitative data usually answers questions like *What*,* How* and *Why*. The qualitative data used in the research project as a whole have been previously Thematically analyzed [42] and reported elsewhere [43] with coding schemes for selected themes. For this paper, subsets of the open-ended responses were selected based on their co-occurrence with respondents’ unexpected two-dimensional response combinations and studied in detail. For example, if a participant responded that they often behaved in an uncivil manner and were satisfied with it, that individual’s open-ended responses were examined to explore possible explanations. The analyzed open-ended responses stemmed from more general questions about incivility (see Appendix A) and were thus *not* directly solicited to explain the specific two-dimensional response combinations. The open-ended answers therefore required some careful categorization and interpretation, to provide relevant insights without incorrectly inferring causation. The qualitative coding was conducted by one of the authors who has extensive experience in qualitative research studies, and the results were discussed among the authors. Few analytical disagreements occurred amongst the authors due to the top-down selection of quotes coupled to specific combinations. This reduced the amount of relevant qualitative data units to a rather limited proportion of the entire dataset. It should be noted that although responses to these questions may point to possible explanations and justifications at individual and contextual levels, they are not to be considered as generalizable. Rather, they can be regarded as indications of the range of respondent group’s experiences.

Not all open-ended responses were clearly comprehensible, e.g., due to language errors (incomplete sentences, unclear wording, inaccurate spelling and grammar, opaque references to previous answers, etcetera). Consequently, the number of responses reported in this study should be regarded as approximate and the value of offering direct quotes should be viewed as providing insight into the respondents’ individual perceptions.

#### Factor analyses

To assess construct validity, confirmatory factor analyses using principal axis factoring and varimax rotation were conducted on all items of the Social Encounters Questionnaire and Straight Forward Incivility Scale. The satisfaction dimensions of each item were analyzed accordingly. Reliability was assessed using Chronbach’s α.

### Ethical approval

All participants provided their written informed consent by checking the consent box prior to accessing the web-based survey. The study was approved by the Swedish Ethical Review Authority, protocol number 2019 − 01513.

## Results

### Study population

732 (72% (77% valid percent)) of the participants were women and 215 (21% (23% valid percent)) men, 1 person (0.1%) stated “Other” as gender; 66 (7%) failed to report gender. Mean age was 41 years (± SD = 12.3 years), while median age was 39 years; age range 18–75 years. 712 respondents (75%) were 31 years or older and 807 (85%) out of 953 (66 missing) had worked at their current workplace for a year or more. Hence, most respondents had sufficient comprehension about their workplace. This is important for the validity of the responses.

### Construct validity

Factor loadings and Chronbach’s α for each subscale are presented in Table 1. Chronbach’s α for the frequency and satisfaction dimensions of civility ranged between 0.603 and 0.899 for all scales. The corresponding results for the incivility dimensions ranged between 0.648 and 0.946. In general factor loadings and Chronbach’s α indicated acceptable construct validity and internal consistency, albeit individual factor loadings for some few items were not sufficient to have been included in indices, if they would have been used in the current study.

**Table 1:**
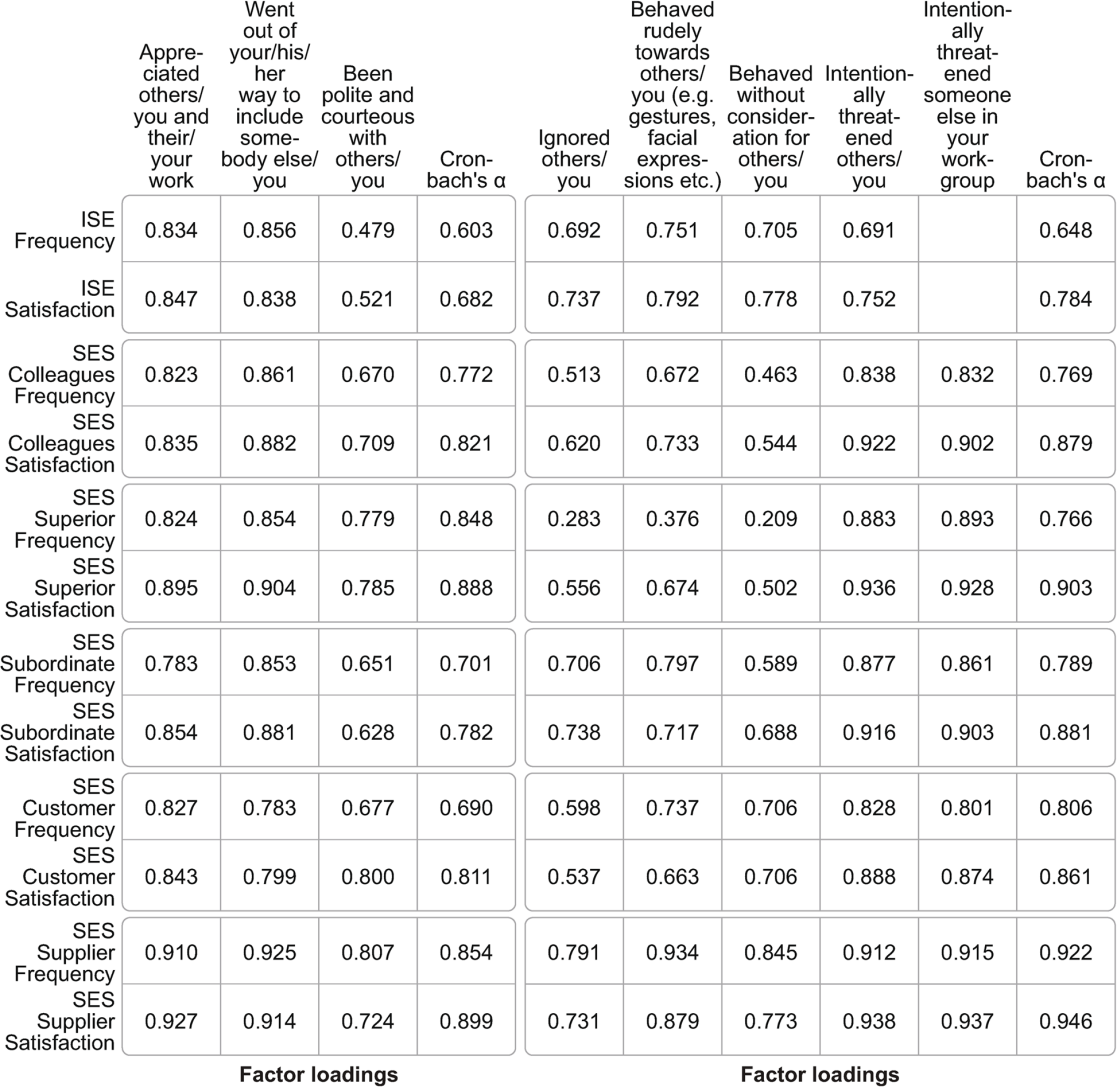
Factor loadings and Chronbach’s α from the confirmatory factor analyses of the slightly modified and two-dimensionally assessed Straightforward Incivility Scale and Social Encounters Scale

### Prevalence of incivility

As has been reported in a previous publication [21], out of 798 people who responded to the single item about prevalence of incivility in the workplace, 62% reported that it occurs, 31% that it does not, and 7% that they do not know. Of the 493 who reported that incivility occurs in the workplace, 404 (82%) reported that they had been exposed themselves, and 419 (85%) had witnessed someone else being exposed to incivility or disrespectful behaviors. About 24% of the open-ended responses concerned experiences of customer incivility (324 out of 1336 responses). This can be contrasted to approximately 20 (1.5%) reporting incivility from superiors and about 27 (2%) reporting incivility between colleagues.

### Rating of own civil and uncivil behaviors

Figure 2 illustrates that 80–96% of the responders rated their own behaviors towards others as often being civil. Most, that is, 71–88%, expressed satisfaction with this. At the same time 1% were dissatisfied with often having shown appreciation for others as well as exerting themselves to include someone else. Except for polite and courteous behaviors, some participants expressed dissatisfaction with every frequency of own civil behaviors. This means that some expressed dissatisfaction if they, for instance, often expressed appreciation for others and their work, while others if they neither often nor rarely, or rarely did it. A similar pattern was demonstrated for the items assessing various dimensions of incivility (Fig. 2).

As expected, most participants responded that higher frequencies of own civil behaviors and lower frequency of uncivil behaviors were satisfactory. At the same time, for both own civil and uncivil behaviors, sometimes the expected satisfying outcome was expressed as being dissatisfying and vice versa. Some individuals seemed to be dissatisfied with high, medium and low frequencies of both some civil and uncivil behaviors. For instance, 1% were satisfied with often ignoring others, while 1% were dissatisfied with often appreciating others and their work. These results, together with the generally wide distribution of results in the crosstabs, indicate the benefits of two-dimensional assessments for a more nuanced interpretation of both civility and incivility.

### Immediate colleagues’ civil and uncivil behaviors

A majority (57–84%) reported that their immediate colleagues often had behaved in civil manners towards them during the past 4 weeks. Most (54–79%) were also satisfied with this. 1–3% stated that their colleagues rarely had behaved in a civil manner towards them and were satisfied with it, while 2–10% stated that their colleagues rarely had exhibited civil behaviors towards them and are dissatisfied with it (see Fig. 3). The wide distribution of responses in the graph once again reflects the complexity of the questions. Civil behaviors are not always perceived as something unequivocally positive. Some (1–3%) were obviously satisfied, or rather tolerated, that their colleagues rarely exhibited civil behaviors.

When it comes to incivility, 78–97% reported that their immediate colleagues rarely had behaved rudely towards them during the past 4 weeks. Most (75–87%) were satisfied with this. 1–7% stated that their colleagues often had exhibited uncivil behaviors towards them and were dissatisfied with it. These results correspond to the outcomes of the questions about collegial civility. The responses were widely distributed for these questions as well (Fig. 3), and certain combinations (see Appendix A) were explored further in the open-ended answers.

### Immediate superior’s civil and uncivil behaviors

A majority (55–80%) reported that their immediate superior often had behaved in a civil manner towards them during the past 4 weeks. Some, 8–25%, responded that their immediate superior rarely had exhibited civil behaviors towards them, 5–19% were dissatisfied with it (Fig. 4).

Conversely, 79–98% reported that their immediate superior rarely had behaved in uncivil ways towards them during the past 4 weeks (Fig. 4). Between 3% and 4% were dissatisfied with this, while 1% responded that their immediate superior often had been rude towards them and were satisfied with it. At the same time, 1–8% were dissatisfied with their immediate superior often exhibiting uncivil behaviors towards them (Fig. 4).

### Subordinates’ civil and uncivil behaviors

In line with the results for own, collegial, and superior behaviors, most subordinates (43–90%) were reported to often behave in civil manners toward their manager during the past 4 weeks. The deviation from this pattern regards the question about subordinates showing appreciation for the manager and their work, where fewer did so compared to own, collegial, and immediate superior’s behaviors. Some, 4–27% responded that their subordinates had rarely exhibited civility toward them, 3–13% were dissatisfied with it (Fig. 5). 85–99% reported that their subordinates rarely exhibited incivility towards them during the past 4 weeks. Between 1% and 6% were dissatisfied with this, while 1% responded that their subordinates often had behaved without consideration for them and were satisfied with it. At the same time, 3–5% were dissatisfied with their subordinates often exhibiting uncivil behaviors towards them (Fig. 5). There was also a wide distribution of responses in the crosstabs for this question, indicating the need for a more nuanced interpretation of the civility and incivility constructs.

### Customers’ civil and uncivil behaviors

The highest levels of dissatisfaction were in general related to customer behaviors. Altogether, 9–34% were dissatisfied with various frequencies of civil and uncivil customer behaviors. While 35–78% often exhibited civil behaviors during the past 4 weeks, 10–12% rarely did so, and 5–7% were dissatisfied with it (Fig. 6). 56–91% reported that their customers rarely exhibited incivility towards them during the past 4 weeks. Between 6% and 11% were dissatisfied with this, while 1% responded that their customers often had behaved without consideration for them and were satisfied with it. At the same time, 3–18% were dissatisfied with their customers often exhibiting uncivil behaviors towards them (Fig. 6). The response distribution in the crosstabs was clearly largest for this question compared with the results for own, collegial, superior and subordinate behaviors. This further supports the two-dimensional approach and importance of a more nuanced interpretation of the civility and incivility constructs.

### Suppliers’ civil and uncivil behaviors

In general, very few (1–4%) were dissatisfied with various frequencies of civil and uncivil supplier behaviors. Whereas 13–40% stated that suppliers often exhibited civil behaviors during the past 4 weeks, 19–36% stated that they rarely did so, but only 1–3% were dissatisfied with it (Fig. 7). 63–79% reported that suppliers rarely exhibited incivility towards them during the past 4 weeks (Fig. 7). Clearly, supplier behaviors seem to be related to more indifference with regards to how satisfied or dissatisfied respondents were with them.

### Insights from the open-ended responses

**Fig. 8:**
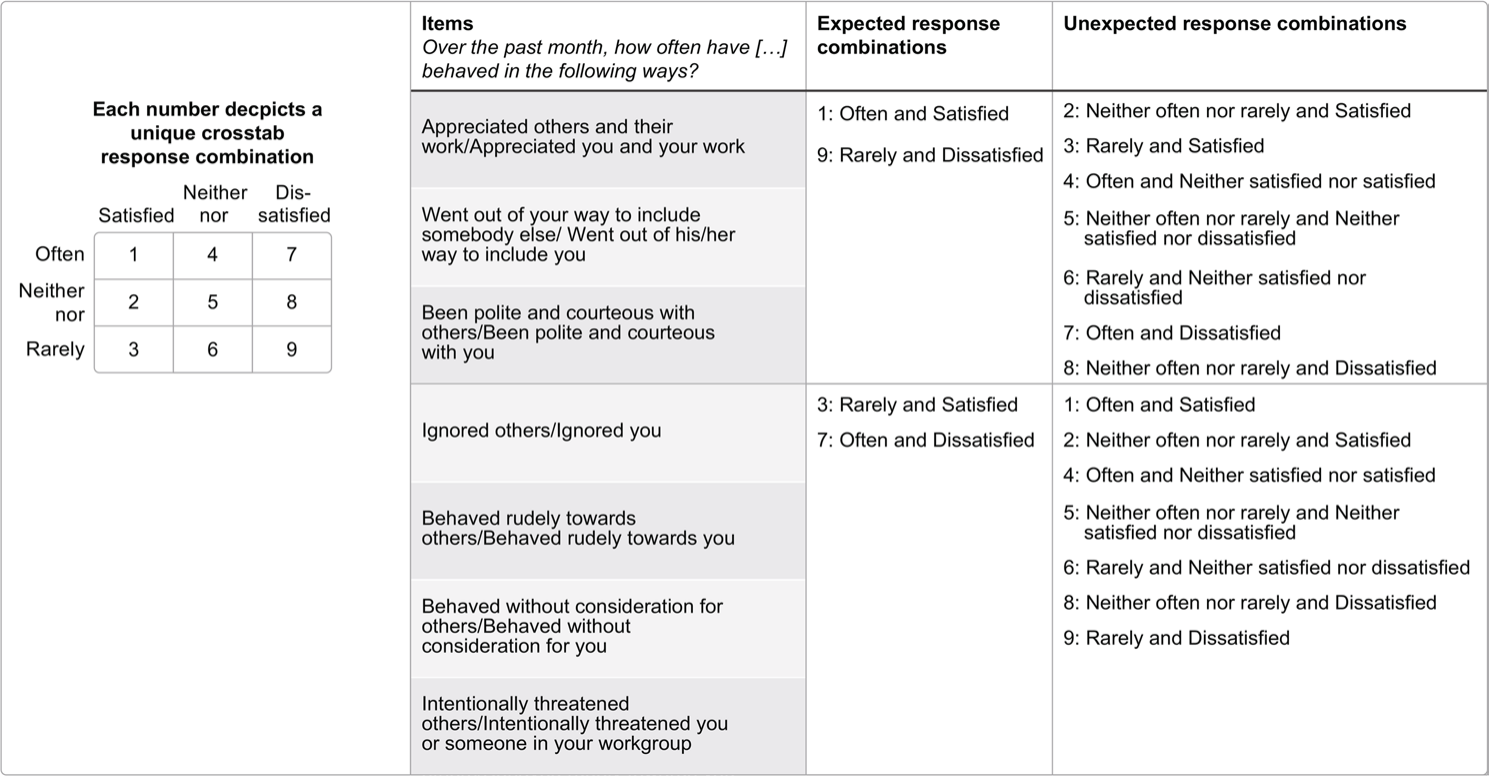
Depiction of the crosstab (to the left). Each number illustrates a specific response combination. The right table demonstrates expected and unexpected response combinations for each item of the Social Encounters Questionnaire and Straight forward Incivility Scale

Most of the two-dimensional response combinations in the crosstabs were expected and in line with the underlying theory behind the Social Encounters Questionnaire [24] and the Straight Forward Incivility Scale [40, 41] used in this study. However, some combinations were unexpected (see Fig. 8). It is not intuitively nor theoretically clear how to interpret the combinations of, for instance, rarely being polite and courteous with others and being satisfied with it (1%) or rarely ignoring others and being dissatisfied with it (5%). Albeit such combinations are unexpected, they may have reasonable and contextual explanations, and do not necessarily imply ill intent.

To better understand the unexpected response combinations, they were further explored qualitatively. Responses to the available open-ended questions such as “*How do you / your immediate colleagues / immediate superior / organization deal with incivility when it occurs?*” were selected for analysis based on their connection to unexpected combinations in the crosstabs. As demonstrated in Appendix A, the open-ended questions did not explicitly ask the respondents to explain their responses to the two-dimensional questions. However, they may still provide some insights to the underlying reasons for the unexpected response combinations. Indeed, the qualitative analyses revealed partial support for the need of a more nuanced interpretation of the civility and incivility constructs. This will be illustrated by quotes associated with the unexpected response combinations presented below.

#### Ignoring others or being ignored by others

Ignoring others or being ignored by others, is theoretically considered as uncivil behavior [24] and was thus expected to be dissatisfying if it occurred. However, the crosstabs revealed that some participants were satisfied or neither satisfied nor dissatisfied with such behavior. The open-ended responses provided some possible explanations. Quotes by 40 participants were identified that could provide potential explanations for this unexpected finding. The quotes indicated that ignoring others could be a way to handle or cope with incivility. For example, one participant explained: *“I ignore [incivility when it occurs] and talk to my partner about it. [My colleagues] ignore it or talk to other colleagues”.* Another participant stated: *“I try to focus on other things [when exposed to incivility]”.*

Indeed, ignoring stressful events, such as incivility, can represent various coping strategies [44, 45]. For example, a retail worker who ignores a customer who behaves in a rude manner may use coping strategies such as avoidance, restraint, disengagement, denial, or acceptance. According to Cortina and Magley [46], the most common coping strategy when exposed to incivility at work is ignoring the person who is being uncivil. This is seemingly not a successful strategy for counteracting future disrespectful behaviors [47]. Still, two participants explicitly stated that ignoring others may be a successful strategy to avoid escalating a fairly mild uncivil situation, which is exemplified with the quote: *“We try to turn the other cheek and make sure that the [uncivil] situation does not become worse. We listen and try to understand why the customer is rude”.* This de-escalating strategy is also described by Cortina & Magley [46], and is in line with restraint coping described by Carver et al. [44]. Restraint coping entails controlling one’s immediate impulses and avoiding behaviors or actions that might risk worsening the situation. Thus, the crosstabs and open-ended responses support the two-dimensional notion that *ignoring others* is a construct that can mean different things for different individuals and in different situations. So, depending on the context, ignoring others when being exposed to incivility from others can for instance represent an uncivil behavior or a coping strategy.

#### Being polite and courteous

For those who exhibited the unexpected response combination of rarely being polite and courteous with others and being satisfied with it, 8 quotes were identified that may elucidate this outcome. For example, one respondent wrote: *“I tell [the uncivil party] that it’s not OK to act like that”.* Another stated: *“I try to put my foot down*,* but it’s usually a precarious situation and you feel vulnerable. [I] don’t get any support from colleagues when it happens. They all just watch or walk away”.* The descriptions of speaking up and setting boundaries could indicate that being polite and courteous is not appropriate in all situations, for example when faced with uncivil behaviors by others. Therefore, satisfaction with rarely being polite and being satisfied with it can indicate contentment with preserving own integrity in hostile situations. This is in line with the “tit-for-tat” attitude described in the literature, implying that exposure to incivility tends to trigger uncivil behavior in return and creates a spiraling effect [5, 15, 48]. Thus, the open-ended responses seem to provide some possible explanations for the unexpected response combinations, which consequently partially supports the need for a more nuanced interpretation of the civility and incivility constructs. The results imply that some feel the need to act in a way that intuitively may seem uncivil but is perceived to be appropriate behavior under certain circumstances.

#### Customers often behaving rudely

For those who displayed the unexpected response combination of being satisfied or neither satisfied nor dissatisfied with customers often behaving rudely, 28 open-ended responses provided some insights. Several participants mentioned that uncivil and rude behavior from customers is common, and five participants reported that having support from their colleagues and manager helped them through uncivil situations. One participant stated: *“With the customer who is uncivil you just have to smile and get through the situation (…). Afterwards*,* you may need to ventilate and talk about the event with a colleague/manager”.* This indicates that customer incivility is, to some extent, expected and that acceptance as well as collegial and managerial support may act as a buffers and a coping strategy when exposed. Another participant stated: *“Customers acting rudely occurs every day in retail. I usually don’t take it to heart so much”.* Another quote exemplifying this was: “*Take it with a pinch of salt (…) and with your actions [you can] show how politeness and civility can give positive energy!”* Thus, the participants’ active coping strategies, such as trying to change a situation for the better, and their attitudes toward the behaviors, and acceptance might explain some of the responses. These results partially support the importance of a more nuanced interpretation of incivility, indicating that some may not necessarily be as bothered by it as others.

#### Rarely receiving appreciation for work

Among those who reported being satisfied or neither satisfied nor dissatisfied with rarely receiving appreciation at work, 69 quotes were identified that could potentially provide some explanations for these unexpected response combinations. Most participants described situations where the customers’ behaviors were understandable to some extent. For example, one participant described: *“[I] try to be nice and polite [when someone is uncivil] and imagine that they are having a bad day or might be ill. But sometimes it’s difficult and you become angry*,* sad and drained of all energy”.* Thus, whereas some individuals may consider lack of appreciation as an uncivil behavior, others may perceive the same behavior to be expected, logical and understandable in certain situations. They rationalize that the uncivil behaviors are not necessarily personal, and, albeit perhaps energy draining, adopt a compassionate attitude instead of being offended.

In summary, apart from the possibility that some participants may have responded incorrectly, some open-ended responses further strengthen the notion that two-dimensional assessments of civility and incivility can increase the nuance and interpretability of the constructs. Some may perceive apparent uncivil behaviors as something expected, appropriate, excusable, or defensible under certain circumstances. Others describe ways to emotionally detach using various forms of active (e.g., taking action to improve the situation, acceptance) or passive (e.g., ignoring or avoiding) coping strategies and/or through support from colleagues and/or manager. Thus, satisfaction or dissatisfaction might in this case not necessarily indicate preference or tolerance of such behaviors but may rather indicate a variety of coping strategies.

## Discussion

The aims of the current mixed methods study were to investigate the past 4-week prevalence and frequency of civility and incivility in a sample from the Swedish retail sector, and to assess a possibly more nuanced interpretation of the civility and incivility constructs using the combination of unique, two-dimensional assessments and open-ended questionnaire responses. To our knowledge, this is the first study to utilize two-dimensional assessments of civility and incivility, and the results show that the appraisal dimension can yield more nuanced interpretations for both constructs. This conclusion is further reinforced by analyses of the open-ended questions, that provide some clarifications for the unexpected combinations found in the two-dimensional assessments.

Even if ‘one size’ does not seem to fit all, most respondents were, as expected, satisfied with high frequency of civility and low frequency of incivility. However, most crosstabs also demonstrated a wide distribution of outcomes. Thus, certain results were more unexpected, such as some expressing satisfaction with high frequency of incivility and low frequency of civility. At the same time, some responses indicated unclear or varying levels of (dis-)satisfaction with, or indifference to, exposure to different frequencies of civility and incivility.

### Prevalence of incivility

The questions about civility and incivility were formulated to cover the past 4 weeks. When using the frequency dimension of the SES-item “Behaved rudely towards you” as a general indicator of incivility, the prevalence was 60%. The reported incivility from colleagues, that is, often and neither nor, was 7% (Fig. 3: 2 + 5), managers 11% (Fig. 4: 4 + 7), and customers 42% (Fig. 5: 22 + 20%), that is, a total exposure of 60% (7 + 11 + 42). The incivility exposure from subordinates and suppliers were excluded, since these figures were not applicable for all participants. This figure corresponds to previously presented results from the current study [21], where a direct question in present tense was utilized to assess incivility prevalence. There, 62% indicated that it occurs, and the nearly identical outcomes may indicate that the current situation strongly influences assessments of the past 4 weeks. This conclusion is supported by previous research indicating that the current situation influences retrospective ratings [49–52]. So, the difference between the results of the current and previous studies is probably explained by differences in assessment time spans.

Further evidence for this conclusion is that Leiter et al. [24] found that the Social Encounter Scale, which this item was derived from, is only moderately correlated when used 6 months apart. Consequently, assessments of incivility prevalence during the past 4 weeks seems, to some extent, vary over time. If this is true, it implicates a potential validity issue in previous research, using retrospective assessments covering the past year [23, 53, 54] or five years [11], for instance. If incivility ratings fluctuate over time, long-term retrospective ratings may yield misleading results since it is not possible to assess the past year or years in a reliable and valid manner. However, assessments of the current situation and the past 4 weeks seem to yield nearly identical outcomes, which implies that these time spans may be used interchangeably.

### Possible explanations for unexpected results

There are several possible explanations for the unexpected response combinations found in this study. Some may be due to different interpretations of the questions, social desirability, context-specific coping or just erroneous scoring. However, given the systematically wide distributions of responses in the crosstabs, it is clear that the constructs and experiences of civility and incivility are indeed multifaceted. This notion is further supported by the open-ended responses.

The crosstabs imply that low frequency of civility and high frequency of incivility is not always perceived as something unequivocally dissatisfactory. Some respondents (1–3%) were for instance apparently satisfied with their colleagues rarely exhibiting civil behaviors. At the same time, 1% stated that their customers often were polite and courteous and were dissatisfied with it, while 1–4% stated that their customers rarely displayed civil behaviors and were satisfied with that. This indicates that a few individuals may view frequent prevalence of civility as a problem, and some that rare prevalence is desirable.

Given our limited capacity to control and adapt our behaviors, previous literature has found that self-regulation can be strenuous [55, 56]. Civil behaviors associated with the retail and service sector could, for instance, mean that some individuals feel compelled to behave in ways they do not feel comfortable with. For example, some participants clarified that ignoring uncivil customer behaviors may sometimes imply adequate or professional conduct, which means that they might have to smile and be polite against their will. Constantly and actively making efforts to control one’s behaviors, feelings and impulses can be stressful and lead to ego depletion. The repeated stress of regulating own behaviors, managing other forms of stress as well as negative emotions can be unreasonably draining, with learned helplessness and stress-related illnesses as possible consequences [56, 57]. Similarly, civil behaviors can also be perceived as “artificial” or “inauthentic” by some under certain circumstances, such as when being exposed to incivility. In such cases, civility would be perceived as dissatisfactory and detrimental. This perspective is further strengthened by individual differences. For example, individuals who score low on the personality trait agreeableness can act in a way that may be perceived as uncivil by others [48]. For them, however, it may be habitual to act in an argumentative and hostile manner, and some even argue that a culture of civility can suppress their ability to voice opinions [58].

Conversely, some individuals indicated dissatisfaction with various forms of incivility rarely occurring. This could be as simply explained as the respondents perceiving any frequency of incivility as bothersome, even if it rarely occurs. Or, perhaps it is an indication that they, for various reasons, would prefer these uncivil behaviors to be more frequent. For instance, 5% were dissatisfied with rarely ignoring others. Again, this could indicate that even rare occasions are unacceptable, or possibly a desire to disregard or not pay attention to persons (customers) who are uncivil. Although ignoring others is theoretically a construct of incivility [24], it is also a well-established coping strategy [44–47]. This implies that, if unidimensional assessments are utilized to assess civility and incivility, it is unclear whether frequently ignoring others is desirable or undesirable. If considered to be an uncivil behavior, it may be undesirable. However, if considered to be a coping strategy, it may be either desirable (if perceived to be effective and an active form of coping) or possibly undesirable (if perceived to be ineffective or a passive or avoidant form of coping).

The “neither satisfied nor dissatisfied” category also needs to be addressed. These responses can imply that the respondents are sometimes satisfied with the selected frequency of civility/incivility and sometimes dissatisfied. They could also signal indifference or indicate a non-response. Some respondents may for instance not know how to respond or did not want to express their opinion. There may be several other possible explanations for the neither-nor-responses. Certainly, the implications of these responses for health- and work-related outcomes will be further analyzed in a future study. A recently published study using two-dimensional assessments of workload demonstrates that the satisfaction and dissatisfaction dimensions are profoundly, systematically, and repeatedly related to indicators of health, wellbeing and the psychosocial work environment, measured by weekly assessments for over two years [25]. The frequency dimension did not exhibit any substantial effects on the same outcomes. The outcomes for the neither-nor category were consistently in-between the satisfied and dissatisfied categories. This indicates that the satisfaction-dissatisfaction dimension is important for actual outcomes, and that the neither-nor category, could in some sense be classified as the “medium” risk level with regards to outcomes, irrespectively of what the response means.

In summary, the two-dimensional assessments of civility and incivility in the current study clearly highlight that one size does not fit all and that the civility and incivility constructs could indeed benefit from a more nuanced assessment approach. Although the majority prefer civility, some are bothered by it and are satisfied if it rarely occurs. Working in retail constitutes a service profession, and several open-ended responses indeed demonstrated signs of service-mindedness, and that civility is a key component of the study participants’ work ethics. However, the results also indicate that this is not the case for everyone and that dissatisfaction risks becoming a problem for individuals, teams, customers and possibly also organizations. There may be a need to regularly examine expectations and needs regarding civility and incivility and to establish routines for managing situations where dissatisfaction is noted, regardless of how often or rarely civility or incivility occurs. Furthermore, when assessments are applied in practice, facts-based dialogues about the results are crucial for correct interpretations of results. Without such dialogues, interpretations may be misleading and yield interventions that may do more harm than good for some individuals or groups.

### Methodological considerations and practical implications

To begin with, since this is a cross-sectional study, causality cannot be inferred. The main focus of this paper was not only the actual outcomes, but also to obtain a better understanding of the constructs of civility and incivility when adding an appraisal dimension. Therefore, the possible effects of confounders such as age, gender, personality, etcetera, have not been considered. These may fully or partially explain some of the outcomes. Also, contextual factors may have a stronger association with incivility than demographic and personality factors [10]. This topic will be fully addressed in future publications where more in-depth analyses will be conducted. The two-dimensional assessments and more detailed analyses will provide a more nuanced understanding of the personal and organizational conditions that are crucial for civility and incivility to relate to positive and negative health- and work-related consequences. This knowledge will be important when it comes to pedagogical development of interventions to counteract various forms of incivility and to actively promote civility. Such knowledge can mean that some employees become aware of their own expectations of others’ behaviors, which can be a problem, while others need to learn what is perceived as uncivil.

As mentioned above, the questions about civility and incivility were formulated to cover the past 4 weeks, whereas most studies about incivility inquire about past year or five years. The findings, which reflect a lower prevalence of incivility compared to previous studies [16–20, 59], are further reinforced by the fact that civil behaviors seem to be common. For example, 80–96% reported that they themselves often exhibited civil behaviors towards others, and 57–84% and 55–80%, respectively, that their colleagues and immediate superior often behaved in civil manners towards them (Figs. 2, 3 and 4). 35–78% of the customers were also reported to often display these forms of civil behaviors (Fig. 6). Only 1–2% stated that they themselves often exhibited uncivil behaviors, while 88–99% stated that they rarely / never did it (Fig. 2). The same applied to the behavior of colleagues, where 1–8% often displayed different forms of incivility, but most, 78–97% rarely did so (Fig. 3). Uncivil behaviors seem to be slightly more common among immediate superiors, where 1–9% often display them, and 79–98% rarely or never do so (Fig. 4). Those who above all seem to exhibit uncivil manners are customers, where 3–22% are reported to behave in uncivil ways often, and 56 − 91% to rarely or never do it.

For a unidimensional approach, any prevalence of incivility may be considered important. In this regard, our prevalence figures resemble previous studies more. Also, it makes sense that incivility, on average, is related to negative outcomes. But it does not mean that these associations are applicable for all contexts, which is clearly demonstrated by the two-dimensional assessments and the open-ended responses. As mentioned above, prevalence differences between the current and previous studies are probably partly explained by the different time intervals, which has been indicated by a previous study comparing different timeframes for incivility questions [60]. Here we inquire about civility and incivility during the past 4 weeks, while other studies have selected the past year or five years. There are advantages and disadvantages to having chosen the past 4 weeks as the timeframe. One of the advantages is reduced risk for recall bias, since it is easier to remember and thus respond more correctly. At the same time, the past 4 weeks may not be representative of the usual situation. This notion is however partly contradicted by Matthews & Ritter [60] who, based on their findings, suggest that the past month seems to be the most appropriate cognitive frame of reference or snapshot of working conditions and attitudes. Shorter timeframes may thus underestimate the prevalence of civility and incivility at work from a more general perspective or overestimate it if the situation has been unusual. At the same time, long timeframes such as one or five years are more prone to recall bias and probably overestimate the prevalence or significance of incivility. It is unlikely that incivility would not have occurred during such a long period of time, especially in the retail sector, and it is furthermore impossible to correctly recall the past year or five years, which makes the responses less reliable. Moreover, as the current study indicates, all prevalence of incivility does not have to constitute a problem. One or more uncivil incidents during the past year or years can already be psychologically processed, while incidents that occurred during the past 4 weeks may not be. In other words, assessing the past 4 weeks can to a greater extent account for current or recent issues, which are also likely to significantly impact other results. An ongoing conflict, for instance, would be more burdensome than one that was ongoing or ended one or two years ago. Taking these aspects into account, and with guidance from the two-dimensional assessments, the advantages of inquiring about the past 4 weeks outweigh the disadvantages.

#### Low response rates

In the methods section we elaborated on possible explanations for the low response rates since the study generated far fewer participants than expected. Furthermore, about half of the participants responded to the questions included in the current paper (Fig. 1). This raises questions as to possible selection and attrition bias and consequently about the generalizability of the results. However, with regards to demographics, mean age and gender distribution was basically identical between the total sample and those included in the current analyses. The attrition was approximately 40% for both genders. Since only managers obtained the questions about subordinates the response rate is 12%, which constitutes about half of the eligible managers, i.e., similar response rates for managers as for the rest of the sample. Given that every question was analyzed and presented separately, there was a negligible variation in the number of responses to each of the civility and incivility question areas (i.e., own, colleague, superior, customer, etc.). In summary, based on age and gender, the responders did not differ from the total sample that also included the non-responders. Consequently, the results seem to be representative of the whole sample. The prevalence of incivility was slightly lower than previous studies, which may be explained by the four-week retrospective rating in the current study, compared to the past one or five years in previous studies. So, we assess that the risk for selection bias is low and that the results are generalizable.

#### Limitations of open-ended questions in surveys

The length and the comprehensibility of open-ended responses varied greatly, and the researchers’ interpretations may have been limited by face-value understanding, bias or language issues. Since the surveys excluded any further opportunity to seek clarification or elaboration from the respondents, a series of interpretive decisions were needed to meaningfully condense and curate the content. For example, an initial, basic filtering of the open-ended responses was conducted to screen out non-meaningful responses (see Appendix A) before addressing the content of good-faith responses. Evidently, using the available open-ended responses did not completely clarify the unexpected response combinations seen in the crosstabs. It would have been optimal to let the participants explain each response combination in the crosstabs, to better understand the underlying reasoning behind them. However, that would have resulted a significantly more extensive questionnaire and would thus have been too time-consuming for the participants to complete. Future studies may consider that approach, or perhaps conducting interviews with a subset of respondents, in order to gain a deeper understanding of the possible multidimensionality of civility and incivility.

## Conclusions

When incivility is assessed for the past 4 weeks, the prevalence is lower than reported in previous studies with retrospective ratings for the past year or more. The two-dimensional assessments of civility and incivility suggest that unidimensional assessments of these constructs may yield misleading interpretations. Although most are satisfied with rare occurrence of incivility, some are dissatisfied with the same. The fact that civility or incivility on average or mostly is associated with positive or negative experiences does not mean that it applies for everyone. This insight becomes particularly important when assessments are used as a basis for systematically ensuring good and healthy work environments. There is undoubtedly a risk that “intuitive” interpretations of unidimensional assessments of civility and incivility are based on theory or preconceived notions rather than accurate assessments. For example, although ignoring others is theoretically considered to be part of the incivility construct, it is also a coping strategy that may be used to handle incivility and hinder it from escalating. Consequently, those with different or “unexpected” experiences, expectations and needs risk being overlooked if unidimensional assessments are used. Future studies should investigate possible confounders (e.g., age, gender, personality, organizational- and situational factors), that need to be considered for a better understanding of whether and when civility and incivility constitute a problem, for whom and under what circumstances. The conclusion of the present study is that civility and incivility constitute constructs that can be interpreted with more nuance when using two-dimensional assessments, and they need to be assessed accordingly for correct and valid interpretation of the results. The perception of a behavior, or the consequence of it, is ultimately what determines whether it was civil or uncivil. Bearing the importance of the appraisal dimension of these constructs in mind will reduce the risk of erroneous conclusions and thereto related erroneous interventions at individual, group and organizational levels.

## Supplementary Information

Supplementary Material 1.

## Data Availability

Data will be made available upon reasonable request to: [dan.hasson@ki.se](mailto: dan.hasson@ki.sedanh) , given that adherence to the General Data Protection Regulation (EU) 2016/679 can be established.
