# Supplementary material for: Two-dimensional assessments of civility and incivility at work
Source: BMC Psychol. 2026 Jun 9;14:846. doi: 10.1186/s40359-026-04927-2 (PMC13251292; doi:10.1186/s40359-026-04927-2)
Supplement: Supplementary file 1 — Supplementary Material 1. [file 40359_2026_4927_MOESM1_ESM.docx]

# **Appendix A – Open-ended survey questions and responses**

The survey as a whole consisted of approximately 300 items in total (some of which were only conditionally asked when respondents answered that they had a particular role). Out of these, eight questions were open-ended, where respondents could elaborate their thoughts. These questions were:

1. How do you deal with incivility when it occurs?
2. How do your immediate colleagues deal with incivility when it occurs?
3. How does your immediate superior deal with incivility when it occurs?
4. How does your organization deal with incivility when it occurs?
5. Does the organization's dealing with incivility lead to any consequences, and if so which ones?
6. How would you want incivility to be dealt with by you / your immediate colleagues / immediate superior / the organization at your workplace?
7. Can you give any examples of how you/colleagues/superior/organization have dealt with incivility in a good way?
8. If you have comments or opinions, please write them here.

Out of 1,014 survey respondents, the open-ended questions were at most answered by 622 respondents (all of whom had answered “Yes” to the question about prevalence of incivility at the workplace). The table below shows the filtering process for the open-ended survey items before subjecting subsets of the remaining meaningful answers to analysis. For abbreviation purposes in the table below, the open-ended questions are labelled A, B, C, etcetera.

| ***Number of excluded…*** | **A** | **B** | **C** | **D** | **E** | **F** | **G** | **H** |
| --- | --- | --- | --- | --- | --- | --- | --- | --- |
| Blanks | 590 | 598 | 605 | 609 | 621 | 336 | 637 | 925 |
| Non-meaningful* responses | 2 | 2 | 6 | 12 | 23 | 22 | 15 | 0 |
| Answers that in their entirety were incomprehensible or obviously insincere | 0 | 0 | 0 | 0 | 0 | 2 | 1 | 0 |
| "Do not know"** (or similar) | 0 | 23 | 68 | 130 | 148 | 107 | 16 | 0 |
| Answers that refer to the content of a previous answer, with no clarifying details | 0 | 48 | 20 | 9 | 1 | 30 | 3 | 0 |
| **Remaining number of responses for analysis** | **422** | **343** | **315** | **256** | **221** | **517** | **342** | **89** |

* Consisting of a single letter, punctuation marks or irrelevant numbers

** Varying wordings, such as “No idea”, “Unclear”, “I don’t know” etcetera.

In this paper, only open-ended responses from participants with specific, “unexpected” response combinations of frequency and satisfaction were analyzed. This means that, of the above available responses, only a relevant subset was included.
